# Pro‐Inflammatory Signaling in Dermal Fibroblasts: A Possible Role of Different Botulinum Toxin Formulations on IL‐6 Expression

**DOI:** 10.1111/jocd.70646

**Published:** 2026-01-02

**Authors:** Sabrina Sommatis, Roberto Mocchi, Serena Di Francesco, Stefano Bighetti, Luca Bettolini, Andrea Carugno, Giovanni Paolino, Mariateresa Rossi, Stefania Guida, Mario Valenti, Nicola Zerbinati

**Affiliations:** ^1^ UB‐CARE S.R.L.‐Spin‐Off University of Pavia Pavia Italy; ^2^ Dermatology Department University of Brescia Brescia Italy; ^3^ Department of Medicine and Surgery University of Insubria Varese Italy; ^4^ Dermatology Unit Università Vita‐Salute San Raffaele Milan Italy; ^5^ Dermatology Clinic IRCCS San Raffaele Scientific Institute Milan Italy; ^6^ Dermatology Unit IRCCS Humanitas Research Hospital Milan Italy; ^7^ Department of Biomedical Sciences Humanitas University Milan Italy; ^8^ University of Insubria, Department of Medicine and Technology Innovation Varese Italy

**Keywords:** BoNT‐A formulations, botulinum toxin type A, complexing proteins, dermal fibroblasts, interleukin‐6 (IL‐6), pro‐inflammatory cytokines

## Abstract

**Background:**

Botulinum toxin type A (BoNT‐A) formulations are extensively employed in aesthetic and therapeutic dermatology. However, their immunomodulatory and pro‐inflammatory properties remain incompletely characterized, particularly concerning dermal fibroblasts.

**Aims:**

This study aimed to evaluate and compare the effects of three commercially available BoNT‐A formulations—Bocouture, Vistabex, and Azzalure—on the expression of interleukin‐6 (IL‐6), a key pro‐inflammatory cytokine, in cultured adult human dermal fibroblasts.

**Patients/Methods:**

Human dermal fibroblasts were cultured in vitro and treated with increasing concentrations of Bocouture, Vistabex, and Azzalure. IL‐6 expression was measured at 24, 48, and 72 h posttreatment using an enzyme‐linked immunosorbent assay (ELISA). Cell viability was assessed by MTT assay to exclude cytotoxic effects. Each condition was tested in triplicate. Data were analyzed using one‐way ANOVA with Bonferroni's multiple comparisons posttest.

**Results:**

Vistabex significantly increased IL‐6 expression at multiple time points, with the most marked elevation observed at 0.5 U/mL after 24 h (*p* ≤ 0.0001). Conversely, Bocouture and Azzalure did not induce significant changes in IL‐6 levels across all tested concentrations and time intervals. No formulation reduced fibroblast viability below 80%, confirming the absence of relevant cytotoxicity.

**Conclusions:**

These findings suggest a formulation‐specific inflammatory response to BoNT‐A in dermal fibroblasts, with Vistabex eliciting a notable upregulation of IL‐6. The observed differences may be attributed to distinct structural or excipient‐related properties and warrant further investigation to clarify their clinical implications, especially in patients with a predisposition to inflammatory skin responses.

## 1 Introduction

Cytokines are small proteins (5–20 kDa) that play an important role in the immune system: in particular, they are able to influence the activation, maturation, growth, and differentiation of several cell populations. Within the large cytokines' family, interleukin (IL‐) 6 plays a crucial role in inflammatory response by representing one of the main acute phase proteins and promoting inflammation in a chronic state. The levels of this interleukin, together with IL‐1 and the tumor necrosis factor alpha (TNF‐α), are high in most inflammatory states, and these cytokines have also been recognized as therapeutic targets. In particular, IL‐6 is involved in the defense against microbiological pathogens. When the body is affected by an infectious episode, a systemic response is activated, characterized by fever, leukocytosis, and changes in the production of various proteins by the liver. This inflammatory response helps the body defend itself from the invasion of the microorganism, and the three cytokines previously mentioned are the major pro‐inflammatory signals generated to elicit this response. IL‐6 is the first that can be detected in serum, and it is mainly responsible for both fever and the acute phase response of the liver [1].

Another fundamental role of IL‐6 is in promoting the transition from an innate and unspecific initial immune response to a more targeted and sustained immune response, through the recruitment of specific leukocyte subpopulations that replace the initial neutrophilic predominant population [2]. Produced by macrophages, but also by different cell lines such as T cells, B cells, monocytes, fibroblasts, keratinocytes, and some cancer cells, IL‐6 mediates its effects through a complex mechanism [3]. In classical signaling, IL‐6 stimulates target cells through the binding of a membrane receptor that binds to the receptor protein gp130, which, in turn, leads to the activation of Janus kinase and the subsequent phosphorylation of tyrosine residues inside the cytoplasmic portion of the gp130 protein itself. A lot of cells express gp130, hence the pleiotropic biological functions of IL‐6, including the promotion of T‐cell proliferation and the differentiation of B cells, megakaryocytes, and macrophages [4]. Thus, measurement of IL‐6 in an in vitro model represents a useful and sensitive tool to assess the anti‐inflammatory effect of a cosmetic product, medical device, and raw materials [5].

Botulinum toxin (BoNT) is a neurotoxic protein produced by Clostridium botulinum, widely used in both aesthetic and therapeutic dermatology. While BoNT is primarily known for its mechanism of action—blocking acetylcholine release at the neuromuscular junction—recent studies have highlighted its impact on immune and inflammatory responses. Experimental data from animal models and clinical studies on patients undergoing facial rejuvenation with botulinum toxin injections have shown a significant increase in IL‐6 levels, along with other pro‐inflammatory cytokines such as IL‐2 and IL‐8 [6, 7, 8, 9]. This suggests that BoNT may induce a transient inflammatory response, potentially linked to tissue repair mechanisms, local immune activation, or neuroinflammation modulation. In the context of dermal fibroblasts, BoNT may influence IL‐6 expression through complex cellular interactions, which could provide valuable insights into its broader biological effects. Therefore, assessing IL‐6 levels in fibroblast cultures treated with different BoNT formulations—Bocouture (Merz), Vistabex (Allergan), and Azzalure (Galderma)—can help better understand its potential pro‐inflammatory or immunomodulatory properties.

The aim of the study is to evaluate the expression of IL‐6 after treatment with the three BoNT type A formulations present on the market through the measurement of interleukin levels in human dermal fibroblasts.

## 2 Material and Methods

### 2.1 Cell Cultures

The cell line used in the assay was a human dermal fibroblast (NHDF‐Ad‐Human Dermal Fibroblasts, Adult, CC‐2511 Lonza). The cell line was grown in complete culture medium in conditions of complete sterility and maintained in incubation at 37°C with an atmosphere of 5% carbon dioxide (CO2).

### 2.2 Cytotoxicity Assay (MTT Test)

MTT test is a colorimetric cytotoxicity assay used to test cell proliferation and viability based on mitochondrial efficiency [10]. MTT (3‐(4,5‐dimethylthiazol‐2‐yl)‐2,5‐diphenyl tetrazolium bromide) is a tetrazolium salt that, in the case of viable cells, is reduced from the highly reducing mitochondrial environment of viable cells by the action of mitochondrial dehydrogenase. MTT reduction leads to the formation of formazan crystals, insoluble in the culture medium but soluble in isopropanol, which gives the typical purple color to the mitochondria of viable cells. In contrast, in suffering or dead cells, lacking active mitochondria, MTT will not be reduced, resulting in a less intense purple color. For the direct relationship between respiration and viability, MTT is considered a good assay to evaluate cell viability [11]. This assay has also been validated for the evaluation of BoNT‐A‐induced cytotoxicity in dermal fibroblasts [12]. For the preparation of the assay, cells were homogeneously seeded in 96‐well plates and incubated at 37°C, with a 5% CO_2_ humidified atmosphere. After 24 h, cells were treated with the tested products (Bocouture and Vistabex) with scalar dilutions ranging from 0.25 to 4 U/mL. The product Azzalure was tested from 0.25 to 10 U/mL, since the concentrations suggested for treatment are higher compared to the other tested products. Untreated cells were used as a control. The test was carried out in three replicates for each dilution. After 24–48 and 72 h of treatment, cells were examined under a phase‐contrast microscope to record changes in morphology, possibly due to cytotoxic effects of the product. Morphological changes such as cell rounding and detachment may indicate early apoptotic responses triggered by BoNT‐A [13]. Then, the culture medium was removed, and cells were incubated with the MTT solution (1 mg/mL) at 37°C for 2 h. Subsequently, the solution was removed and replaced with 100 μL of dimethyl sulfoxide (DMSO), and the absorbance was read at 570 nm wavelength using a microplate reader. Cell survival was calculated by measuring the difference in optical density of the products with respect to the control (untreated cells). The test was carried out in three replicates for each dilution.

### 2.3 Analysis of Interleukin Expression by ELISA Kit

The expression of IL‐6 interleukin after treatment with the product was evaluated in NHDF cells, using an ELISA kit (Thermo Fisher). This method has been widely used in fibroblast cultures treated with BoNT‐A to measure cytokine modulation [14]. To perform the test, cells were seeded in a 96‐well plate and then treated for 24–48 and 72 h at the established concentrations. At the end of the treatment, supernatants were collected and used for coating on a specifically pretreated 96‐well ELISA plate provided by the kit. The standards were reconstituted with ultrapure water and used to build the standard curve. Samples were added to each well in duplicate and the assay was performed according to the manufacturer's instructions. The absorbance was then read at 450 nm using a microplate reader.

### 2.4 Statistical Analysis

Statistical analysis was performed with the one‐way ANOVA followed by Bonferroni multiple comparisons posttest by the GraphPad Prism version 9.0.0 software (GraphPad Software Inc), and differences with *p* < 0.05 were considered statistically significant compared to the relative controls.

## 3 Results

The results obtained are reported in the table and charts containing the measurements of cell viability (MTT assay) and interleukin expression after treatment with the products to be tested, Bocouture, Vistabex, and Azzalure, with respect to the related control in the NHDF cell line.

### 3.1 Evaluation of Cell Viability

NHDF cells were incubated for 24, 48, and 72 h with varying concentrations of the tested products diluted in culture medium to assess their cytotoxicity. The evaluation aimed to ensure that cell mortality did not exceed 30%, a threshold beyond which the treatment would be considered excessively cytotoxic.

**FIGURE 1:**
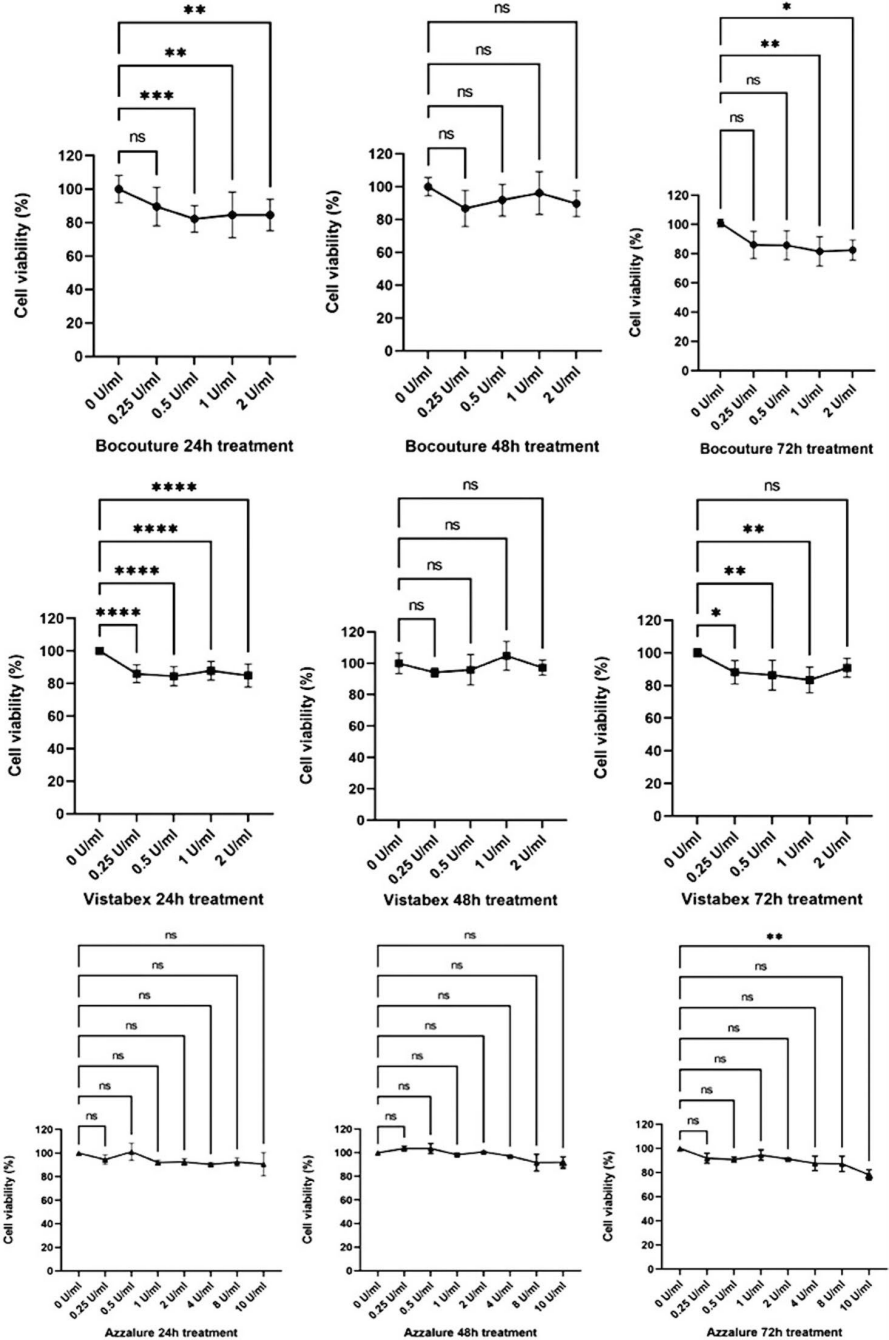
Cell viability after 24–48 and 72 h of treatment of NHDF cells with the tested products. ****p values ≤ 0.0001, ***p values ≤ 0.001, **p values ≤ 0.01, *p values ≤ 0.05 were statistically significant.

In Figure 1, the graph and corresponding data on cell viability, expressed as a percentage relative to the control, are shown as a function of increasing product concentrations after 24, 48, and 72 h of treatment. Bocouture exhibited a statistically significant reduction in cell viability after 24 h, with 0.5 U/mL (*p* ≤ 0.001) and 1 U/mL and 2 U/mL (*p* ≤ 0.01) showing a difference compared to the control. No significant differences were observed at 48 h, while at 72 h, viability was significantly reduced at 1 U/mL (*p* ≤ 0.01) and 2 U/mL (*p* ≤ 0.05). Vistabex induced a greater reduction in cell viability at 24 h, with all tested dilutions reaching statistical significance (*p* ≤ 0.0001). No significant differences were observed at 48 h, whereas at 72 h, viability was reduced at 0.25 U/mL (*p* ≤ 0.05), 0.5 U/mL, and 1 U/mL (*p* ≤ 0.01). Azzalure did not show any significant reduction in viability. The MTT assay confirmed that treatment with the tested products did not lower cell viability below 80%, indicating that cytotoxicity remained within an acceptable range. These results are consistent with prior studies that showed no significant cytotoxic effects of BoNT‐A in dermal fibroblasts under similar conditions [15, 16]. Based on these findings, the highest concentrations were selected for the inflammatory assay.

### 3.2 Evaluation of IL‐6 Expression

The expression of IL‐6 in NHDF cells was quantified using an ELISA kit following treatment with the tested products. Prior in vitro studies have confirmed that fibroblasts exposed to BoNT‐A can exhibit differential IL‐6 responses depending on dose and formulation [17].

**FIGURE 2:**
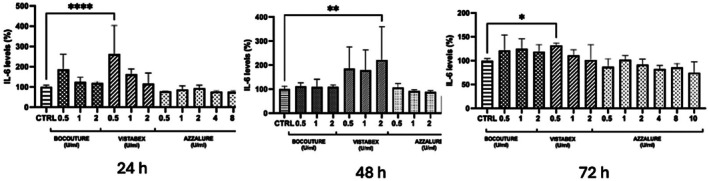
Effect of 24–48–72 h treatment with the products on IL‐6 production, expressed as percentage (*n* = 2; replicates = 2). Ctrl: Untreated cells; 0.5–1–2‐4‐8‐10 U/mL: Treated cells with different concentrations of products. ****p values ≤ 0.0001, **p values ≤ 0.01, *p values ≤ 0.05 were statistically significant compared to untreated cells (Ctrl) (*n* = 2; replicates = 3).

Figure 2 and Table 1 present the IL‐6 levels in NHDF cells treated with the selected concentrations, compared to untreated control cells. The results indicate that Bocouture and Azzalure did not induce an increase in IL‐6 levels under any of the tested conditions. These findings align with the known immunologic differences among BoNT‐A formulations with and without complexing proteins [18]. In contrast, Vistabex significantly upregulated IL‐6 expression at multiple time points: after 24 h (0.5 U/mL, *p* ≤ 0.0001), 48 h (2 U/mL, *p* ≤ 0.01), and 72 h (0.5 U/mL, *p* ≤ 0.05). This observation is consistent with previously reported pro‐inflammatory activity of BoNT‐A in dermal fibroblast cultures [17]. These findings suggest that Vistabex stimulates IL‐6 production in human dermal fibroblasts.

**TABLE 1:** Values of IL‐6 levels, expressed as a percentage, in cells after treatment with the tested products.

Values of IL‐6 levels							
BOCOUTURE (Merz)							
Sample (U/mL)	0	0.5	1	2			
24 h: IL‐6 levels (%) ± SD	100 ± 5.60	187.02 ± 73.89	124.89 ± 22.01	119.31 ± 7.50			
48 h: IL‐6 levels (%) ± SD	100 ± 8.99	111.45 ± 14.94	109.31 ± 31.55	109.50 ± 6.99			
72 h: IL‐6 levels (%) ± SD	100 ± 3.43	121.26 ± 32.66	124.90 ± 20.94	118.65 ± 14.36			
VISTABEX (Allergan)							
Sample (U/mL)	0	0.5	1	2			
24 h: IL‐6 levels (%) ± SD	100 ± 5.60	261.91 ± 141.10	162.39 ± 26.01	115.94 ± 52.79			
48 h: IL‐6 levels (%) ± SD	100 ± 8.99	184.75 ± 91.05	178.55 ± 84.05	221.17 ± 138.93			
72 h: IL‐6 levels (%) ± SD	100 ± 3.43	131.70 ± 5.07	111.02 ± 11.54	101.32 ± 31.84			
AZZALURE (Galderma)							
Sample (U/mL)	0	0.5	1	2	4	8	10
24 h: IL‐6 levels (%) ± SD	100 ± 12.81	78.36 ± 1.18	87.01 ± 18.99	94.47 ± 14.74	76.92 ± 3.73	76.09 ± 4.50	56.77 ± 7.23
48 h: IL‐6 levels (%) ± SD	100 ± 16.58	106.50 ± 17.18	92.16 ± 5.29	88.35 ± 5.07	71.51 ± 7.02	66.88 ± 12.46	58.33 ± 5.96
72 h: IL‐6 levels (%) ± SD	100 ± 5.56	87.09 ± 16.55	101.83 ± 8.73	91.54 ± 11.33	82.71 ± 7.04	85.75 ± 8.28	74.44 ± 23.33

## 4 Discussion

The results of this study suggest that Bocouture and Azzalure do not elicit significant alterations in IL‐6 expression in human dermal fibroblasts compared to the control group. In contrast, Vistabex induces a significant upregulation of IL‐6 expression, highlighting a potential pro‐inflammatory effect. This difference may be attributed to the distinct structural composition, excipient profile, and pharmacodynamic properties of each botulinum toxin type A formulation, which could influence cellular responses and inflammatory pathways [18].

IL‐6 is known to exert diverse biological effects that depend on the physiological context and the degree of its expression. While persistent and uncontrolled secretion of IL‐6 is correlated with harmful inflammatory responses, a transient or low‐level increase may instead facilitate reparative and tissue remodeling processes. Numerous studies have indicated that IL‐6 can enhance fibroblast proliferation and collagen synthesis under specific physiological conditions, including wound healing or regulated inflammatory stimuli [11, 19, 20].

These findings suggest that the moderate increase in IL‐6 observed following Vistabex treatment may indicate not only an inflammatory response but also an adaptive mechanism that could be involved in dermal matrix remodeling. This effect may hold significance in aesthetic dermatology, where controlled activation of fibroblasts and stimulation of the extracellular matrix are regarded as desirable therapeutic objectives [21]. However, this hypothesis warrants further investigation through targeted studies that examine the relationship between IL‐6 modulation and collagen production in both in vitro and in vivo settings [22]. In addition to molecular composition, the differences observed in IL‐6 expression among the various botulinum toxin type A formulations may also reflect the intrinsic characteristics of each product, including the presence of complexing proteins or specific excipients. Although both Vistabex and Azzalure contain accessory proteins, only Vistabex resulted in a significant increase in IL‐6 in our experimental model. This suggests that the mere presence of complexing proteins does not fully account for the inflammatory response; rather, other structural or conformational factors, such as the stability of neurotoxin complexes, protein folding characteristics, and the nature of excipients, may play a critical role in modulating cellular reactivity [23]. Another variable that may account for the differential IL‐6 responses observed among BoNT‐A formulations is the proportion of catalytically active neurotoxin, specifically the 150‐kDa active core comprising the light and heavy chains, within each product. Although clinical units are standardized based on biological activity in mouse models, these units do not reflect the absolute amount of active neurotoxin present. Previous studies have demonstrated that the quantity of active neurotoxin per labeled unit varies significantly among commercial products due to differences in manufacturing processes and purification methods. For instance, Bocouture contains a highly purified 150‐kDa neurotoxin without complexing proteins, and its specific activity has been estimated at approximately 227 U/ng, compared to 137 U/ng for Vistabex and intermediate values for Azzalure [24]. These discrepancies imply that, at the same nominal dose, different formulations deliver varying numbers of active toxin molecules. Moreover, the presence of inactive neurotoxin or protein aggregates may influence receptor binding, endocytosis, and intracellular trafficking, thereby affecting cytokine induction in dermal fibroblasts. In our study, Vistabex, which showed the most consistent upregulation of IL‐6, has been reported to contain a greater total mass of protein, including a higher proportion of accessory and potentially immunogenic components [25]. These compositional differences could modulate local immune recognition or intracellular stress responses, partially explaining the divergent IL‐6 profiles observed in our model. Although IL‐6 was selected as the primary target for this preliminary investigation due to its dual role in inflammation and tissue remodeling, a broader cytokine profile, including IL‐1β and TNF‐α, would offer a more comprehensive immunological characterization. Differences in IL‐6 expression among formulations may result from variations in neurotoxin complex stability, protein folding dynamics, or excipient composition, which in turn influence receptor binding, endocytosis kinetics, and intracellular signaling cascades. These factors may elicit distinct transcriptional responses in dermal fibroblasts.

Intracutaneous diffusion has long been considered a relevant factor in the biological effects of BoNT‐A. However, current clinical and preclinical data suggest that there are no significant differences in diffusion among various formulations. It seems more plausible that the observed differences in inflammatory responses may be linked to factors such as immunogenicity, protein composition, or pathways of cellular internalization. Future studies focusing on receptor interactions, endocytosis kinetics, and the activation of the innate immune system could help elucidate these mechanisms with greater precision [26]. The immunomodulatory effects of BoNT‐A on dermal fibroblasts remain only partially understood. While some studies indicate that BoNT‐A does not significantly alter fibroblast proliferation or cytokine secretion, others have demonstrated its ability to influence fibroblast behavior by inhibiting differentiation into myofibroblasts and modulating extracellular matrix production [12, 13, 27, 28]. These findings support the hypothesis that the cellular effects of BoNT‐A are multifactorial and potentially dependent on the formulation. Utilizing in vitro and ex vivo models with co‐cultures of fibroblasts and immune cells may offer further insights into these mechanisms. It is crucial to highlight that the potentially beneficial effects associated with a moderate increase in IL‐6 are highly context‐dependent, influenced by the biological circumstances and duration of cytokine activation. While acute and transient inflammation may facilitate dermal remodeling through regulated fibroblast stimulation, a prolonged inflammatory response may yield adverse outcomes. From a clinical perspective, the IL‐6 upregulation observed following Vistabex treatment warrants careful consideration, as IL‐6 is a pivotal cytokine in inflammatory processes, associated with edema, erythema, and increased skin rigidity [1, 2, 6, 7, 8, 9, 10, 11, 12, 13, 14, 15, 16, 17, 18, 19, 20, 21, 22, 23, 24, 25, 26, 27, 28, 29, 30]. For patients with predispositions to inflammatory responses, sustained elevation of IL‐6 may lead to dermal fibrosis and pathological remodeling, potentially resulting in clinical manifestations such as hypertrophic scarring or cutaneous sclerosis [6]. This concern is especially pertinent for individuals receiving repeated botulinum toxin injections, where chronic fibroblast activation may promote long‐term adverse effects.

Nevertheless, recent studies suggest that BoNT‐A may also exert antifibrotic properties under specific conditions, such as through the inhibition of the JAK2/STAT3 signaling pathway, which has been shown to reduce fibroblast proliferation and scar formation [31]. Therefore, the overall impact of BoNT‐A on fibroblast‐mediated remodeling likely depends on dosage, formulation, and treatment regimen.

In our experimental model, only Vistabex resulted in a significant increase in IL‐6 expression, while Azzalure and Bocouture exhibited cytokine profiles comparable to the control. These findings support the concept that different BoNT‐A formulations may variably influence inflammatory pathways depending on their structural composition and cellular effects. Consequently, Bocouture and Azzalure, which showed no significant IL‐6 upregulation, may be preferable for individuals with a history of cutaneous hypersensitivity or inflammatory skin conditions. Their simplified formulation may reduce the likelihood of engaging extensive fibroblast populations and triggering unwanted pro‐inflammatory responses.

While this study contributes valuable insights into the effects of botulinum toxin formulations on IL‐6 expression in fibroblasts, several limitations must be acknowledged. The observed IL‐6 response to Vistabex did not follow a clear dose‐dependent relationship: although certain concentrations at specific time points yielded statistically significant IL‐6 increases, the overall trend did not follow a linear or dose‐proportional pattern. This observation may reflect the complex regulatory behavior of fibroblasts in response to external stimuli. In fact, IL‐6 secretion is known to exhibit threshold‐dependent dynamics, oscillatory transcriptional activity, and feedback inhibition, rather than a monotonic or dose‐proportional release. Several studies have demonstrated that IL‐6 signaling follows a biphasic or hormetic pattern, whereby low concentrations may exert anti‐inflammatory or reparative effects, while higher levels promote pro‐inflammatory or fibrotic responses [32]. Additionally, the temporal pattern of stimulation may influence cytokine output: short, transient exposures to pro‐inflammatory signals such as TNF‐α can elicit a more pronounced IL‐6 peak than continuous stimulation, likely due to rapid activation followed by the induction of autocrine inhibitors that dampen further transcriptional activity [33]. Moreover, in vitro data have shown that fibroblasts may exhibit synergistic IL‐6 release only when multiple stimuli cross a critical activation threshold, further supporting the notion of a nonlinear and context‐dependent response [34]. These regulatory mechanisms may explain why only selected concentrations of BoNT‐A formulations elicited significant IL‐6 upregulation in our model, while others did not. Such behavior is further modulated by intrinsic cellular features, including cell cycle heterogeneity, receptor saturation, transcriptional feedback, and interindividual genetic variability, as previously reported in fibroblasts from systemic sclerosis patients and in studies of IL‐6 promoter polymorphisms [35, 36]. The study focused exclusively on IL‐6, without assessing other key mediators such as IL‐1β and TNF‐α, and did not include a positive control for IL‐6 stimulation. In this context, it is noteworthy that Haubner et al. [17] found no significant modulation of IL‐6 expression in fibroblast and endothelial cell models exposed to botulinum toxin. However, important methodological differences exist between the studies, including the use of lower concentrations (0.25 and 0.625 U/mL), shorter exposure durations, and potentially distinct formulation profiles. The use of a monoculture fibroblast model does not fully replicate the complexity of the in vivo environment, where fibroblasts interact with immune cells and extracellular matrix components that influence cytokine signaling. Additionally, the analysis was limited to 24, 48, and 72 h, potentially overlooking earlier or delayed responses. These limitations underscore the need for extended time‐course experiments, broader cytokine profiling, standardized stimulation protocols, and the adoption of co‐culture or in vivo models to better contextualize these findings.

## 5 Conclusions

The findings lay the foundation for the importance of selecting botulinum toxin formulations based on a patient's inflammatory profile and individual risk factors. Further prospective clinical trials focusing on populations predisposed to inflammation will be essential to confirm the long‐term impact of complexing proteins and tissue diffusion on cutaneous immune responses. Additionally, incorporating in vivo models may provide mechanistic insights into how different botulinum toxin formulations interact with cutaneous cells and immune pathways. Such research would contribute to the development of optimized therapeutic strategies that enhance both safety and efficacy in botulinum toxin‐based treatments.

## Author Contributions

S.S.: conceptualization, methodology, writing – original draft preparation; R.M.: conceptualization, methodology; S.D.F.: methodology, software; S.B.: validation, writing – review and editing; L.B.: validation, formal analysis, writing – review and editing; A.C.: investigation, validation; G.P.: investigation, formal analysis; M.R.: data curation, writing – review and editing; S.G.: resources, data curation; M.V.: data curation, visualization; N.Z.: visualization, supervision, project administration.

## Ethics Statement

Ethical review and approval were not required for this study, as it involved only commercially available human dermal fibroblast cell lines (NHDF‐Ad, Lonza), in accordance with institutional and national guidelines.

## Conflicts of Interest

The authors declare no conflicts of interest.

## Data Availability

The data that support the findings of this study are available from the corresponding author upon reasonable request.
